# Structural and Functional Profiling of Environmental Ligands for Estrogen Receptors

**DOI:** 10.1289/ehp.1408453

**Published:** 2014-09-26

**Authors:** Vanessa Delfosse, Marina Grimaldi, Vincent Cavaillès, Patrick Balaguer, William Bourguet

**Affiliations:** 1 Inserm (Institut national de la santé et de la recherche médicale) U1054, Montpellier, France; 2 CNRS (Centre national de la recherche scientifique) UMR 5048, Universités Montpellier 1 & 2, Centre de Biochimie Structurale, Montpellier, France; 3 IRCM (Institut de Recherche en Cancérologie de Montpellier), Montpellier, France; 4 Inserm, U896, Montpellier, France; 5 Université Montpellier 1, Montpellier, France; 6 ICM (Institut régional du Cancer de Montpellier), Montpellier, France; * These authors contributed equally to this work.

## Abstract

Background: Individuals are exposed daily to environmental pollutants that may act as endocrine-disrupting chemicals (EDCs), causing a range of developmental, reproductive, metabolic, or neoplastic diseases. With their mostly hydrophobic pocket that serves as a docking site for endogenous and exogenous ligands, nuclear receptors (NRs) can be primary targets of small molecule environmental contaminants. However, most of these compounds are chemically unrelated to natural hormones, so their binding modes and associated hormonal activities are hardly predictable.

Objectives: We conducted a correlative analysis of structural and functional data to gain insight into the mechanisms by which 12 members of representative families of pollutants bind to and activate the estrogen receptors ERα and ERβ.

Methods: We used a battery of biochemical, structural, biophysical, and cell-based approaches to characterize the interaction between ERs and their environmental ligands.

Results: Our study revealed that the chemically diverse compounds bound to ERs via varied sets of protein–ligand interactions, reflecting their differential activities, binding affinities, and specificities. We observed xenoestrogens binding to both ERs—with affinities ranging from subnanomolar to micromolar values—and acting in a subtype-dependent fashion as full agonists or partial agonists/antagonists by using different combinations of the activation functions 1 and 2 of ERα and ERβ.

Conclusions: The precise characterization of the interactions between major environmental pollutants and two of their primary biological targets provides rational guidelines for the design of safer chemicals, and will increase the accuracy and usefulness of structure-based computational methods, allowing for activity prediction of chemicals in risk assessment.

Citation: Delfosse V, Grimaldi M, Cavaillès V, Balaguer P, Bourguet W. 2014. Structural and functional profiling of environmental ligands for estrogen receptors. Environ Health Perspect 122:1306–1313; http://dx.doi.org/10.1289/ehp.1408453

## Introduction

Endocrine-disrupting chemicals (EDCs) are exogenous substances that interfere with the function of hormonal systems and cause deleterious effects on humans and wildlife (De Coster and van Larebeke 2012; Diamanti-Kandarakis et al. 2009; Schug et al. 2011). Many EDCs are man-made chemicals produced by industry and released into the environment, but some naturally occurring EDCs can also be found in plants or fungi. EDCs can affect the endocrine system of an organism in a wide variety of ways. These include mimicking natural hormones, antagonizing their action, or modifying their synthesis, metabolism, and transport. Moreover, these substances can act via multiple pathways, including membrane receptors, the aryl hydrocarbon receptor, or the enzymes involved in hormone biosynthesis and metabolism (De Coster and van Larebeke 2012). Yet, most of the reported harmful effects of EDCs are ascribed to their interaction with members of the nuclear receptor (NR) family, including the estrogen receptors ERα and ERβ for which a large panel of exogenous ligands have been identified. The group of molecules acting as ER environmental ligands is highly heterogeneous and includes natural phytoestrogens or mycoestrogens, as well as industrial compounds such as pesticides, plasticizers, surfactants, or UV (ultraviolet) filters (Li et al. 2013).

ERs and their endogenous ligand, 17β-estradiol (E_2_), play important roles in the growth and maintenance of a diverse range of tissues. As a consequence, dysfunctional ER signaling (i.e., inappropriate exposure to environmental pollutants) may lead to hormonal cancers, infertility, obesity, or diabetes (Grun and Blumberg 2007; Newbold et al. 2009; Rubin and Soto 2009). ERα is expressed primarily in the uterus, liver, kidney, and heart, whereas ERβ is expressed primarily in the ovary, prostate, lung, gastrointestinal tract, bladder, and hematopoietic and central nervous systems. ERα and ERβ are also coexpressed in a number of tissues including the mammary, thyroid, or adrenal glands; bone; and some regions of the brain. Although ERα and ERβ share similar mechanisms of action, several differences in the transcriptional abilities of each receptor, as well as distinct phenotypes between gene-null animals, have been identified and suggest that these receptors may regulate distinct cellular pathways (Couse and Korach 1999; Curtis et al. 1996). Interestingly, ERβ has been shown to antagonize the effects mediated by ERα on cell proliferation in the breast, uterus, ovary, and prostate (Docquier et al. 2013; Lindberg et al. 2003; Weihua et al. 2000). In this regard, EDCs with selectivity for either ER subtypes may produce different biological outcomes, particularly on cancer cell proliferation.

Like other members of this family, ERs contain three major functional domains, including an N-terminal domain that harbors a transcriptional activation function (AF-1), a DNA-binding domain, and a C-terminal ligand-binding domain (LBD) hosting a ligand-dependent transcriptional activation function (AF-2). The LBD is crucially involved in most of the receptor functions because of its capacity for hormone binding, dimerization, and interaction with coregulators. The LBD also contributes to the modulation of the N-terminal AF-1 through interdomain crosstalk so that both AF-1 and AF-2 domains can recruit a range of coregulatory proteins and act either individually or in a synergistic manner (Kobayashi et al. 2000; Métivier et al. 2001). It is noteworthy that the diversity of transcriptional coregulators mediating the effect of ERs on gene expression combined with variations in their expression levels and posttranslational modifications affect the specificity of the response depending on the cell type, ligand, or target gene considered (McDonnell and Wardell 2010).

**Figure 1:**
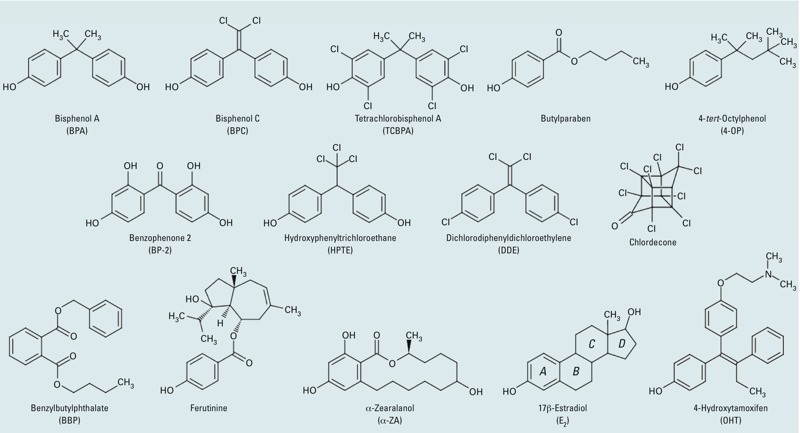
Chemical structures of the natural agonist E_2_, the synthetic antagonist OHT, and the various environmental ER ligands used in the present study.

Our recent work has shown that approaches combining structural, biophysical, and cell-based techniques are helpful in understanding how environmental compounds that are structurally and chemically divergent from natural ligands can interact with NRs and impact their signaling pathways (Delfosse et al. 2012; le Maire et al. 2009; Riu et al. 2011). In the present study, we used a similar approach to gain insight into the mechanisms by which 12 contaminants interact with ERs and modulate their AFs. The compounds used in this study (Figure 1) were selected on the basis of their structural diversity and because they belong to the most representative families of ER environmental ligands. These are bisphenol A (BPA) and bisphenol C (BPC), both used as plasticizers; the flame retardant tetrachlorobisphenol A (TCBPA); the preservative butylparaben; the surfactant 4-*tert*-octylphenol (4-OP); the UV filter benzophenone-2 (BP-2); the pesticides 2,2-bis(*p*-hydroxyphenyl)-1,1,1-trichloroethane (HPTE, a methoxychlor metabolite), dichlorodiphenyldichloroethylene (DDE, a DDT metabolite), and chlordecone; benzylbutylphthalate (BBP); the phytoestrogen ferutinine; and the growth stimulant α-zearalanol (α-ZA), a double reduction product of the mycoestrogen zearalenone.

## Materials and Methods

*Ligands and peptides*. E_2_ (CAS number 50-28-2, purity 98%), BP-2 (CAS number 131-55-5, purity 97%), α-ZA (CAS number 247-769-0, purity 97%), 4-OP (CAS number 140-66-9, purity 97%), HPTE (CAS number 2971-36-0, purity 98), BPA (CAS number 80-05-7, purity 99%), BPC (CAS number 14868-03-2, purity 99%), DDE (CAS number 72-55-9, purity 99%), butylparaben (CAS number 94-26-8, purity 99%), chlordecone (CAS number 143-50-0, purity 99%), and BBP (CAS number 85-68-7, purity 98%) were purchased from Sigma-Aldrich (Saint-Quentin-Fallavier, France). We obtained TCBPA (CAS number 79-95-8, purity 98%) from TCI Europe (Zwijndreccht, Belgium); ferutinine (CAS number 41743-44-6, purity 98%) from Santa Cruz Biotechnology Inc. (Dallas, TX, USA); and 4-hydroxytamoxifen (OHT; CAS number 68392-35-8, purity 99%) from Zeneca (Macclesfield, UK). Compounds were dissolved in DMSO to prepare 10^–2^ M stock solutions. The fluorescein-RHKILHRLLQEGS peptide corresponding to the NR box 2-binding motif of SRC-1 was purchased from EZbiolab (Westfield, IN, USA).

*Reporter cell lines and culture condition*. Luciferase and whole-cell ER competitive binding assays were performed using the reporter cell lines HGELN-ERα, HGELN-ERβ, HGELN-ΔAB-ERα, and HGELN-ΔAB-ERβ as described by Molina-Molina et al. (2008). For details, see Supplemental Material, “Reporter cell lines and culture conditions.”

*Protein production and purification*. The wt-ERα LBD (wild-type) and the ERα-Y537S LBD mutant (amino acids 302–552) were cloned into a modified pET-15b vector, and the wt-ERβ LBD (amino acids 261–502) was cloned into a pET-32a vector. All constructs were expressed in BL21(DE3) cells. Protein domains were purified using a nickel affinity column and size exclusion chromatography, as described in detail in Supplemental Material, “Protein production and purification.”

*Structure determination*. Prior to crystallization assays, the purified ERα-Y537S LBD (final concentration = 0.15 mM) was mixed with 0.3 mM chlordecone, BBP, ferutinine, α-ZA, butylparaben, 4-OP, TCBPA, BP-2, or HPTE, along with 0.3 mM SRC-1 coactivator peptide. Cocrystals were obtained for all complexes in 300–340 mM NaCl, 100 mM HEPES, pH 7.75, and 24–30% polyethylene glycol 3350. Data were collected on the ID23-1, ID23-2, or ID29 beamlines at the European Synchrotron Radiation Facility (Grenoble, France) and processed as described in Supplemental Material, “Data collection and structure determination.”

*Fluorescence anisotropy measurements*.Measurement of the binding affinities of the fluorescein-labeled SRC-1 NR2 peptide (final concentration of 4 nM) for wild-type ERα and ERβ LBDs in both the absence and presence of various ligands was performed using a Safire^2^ microplate reader (TECAN, Männedorf, Switzerland) with the excitation wavelength set at 470 nm and emission measured at 530 nm. The buffer solution for assays was 20 mM Tris, pH 8.0, 150 mM NaCl, 5 mM DTT, and 10% glycerol. All ligands were present at a concentration corresponding to 2 molar equivalents of the highest concentration of protein. The measurements were initiated at the highest concentration of protein (10 μM for ERα or 20 μM for ERβ). The sample was then diluted successively by a factor of 2, with the buffer containing 8 nM of fluorescent peptide and 20 μM or 40 μM of ligand, allowing us to establish the titration curve. Data were analyzed using GraphPad Prism (GraphPad Software Inc., La Jolla, CA, USA).

## Results

**Figure 2:**
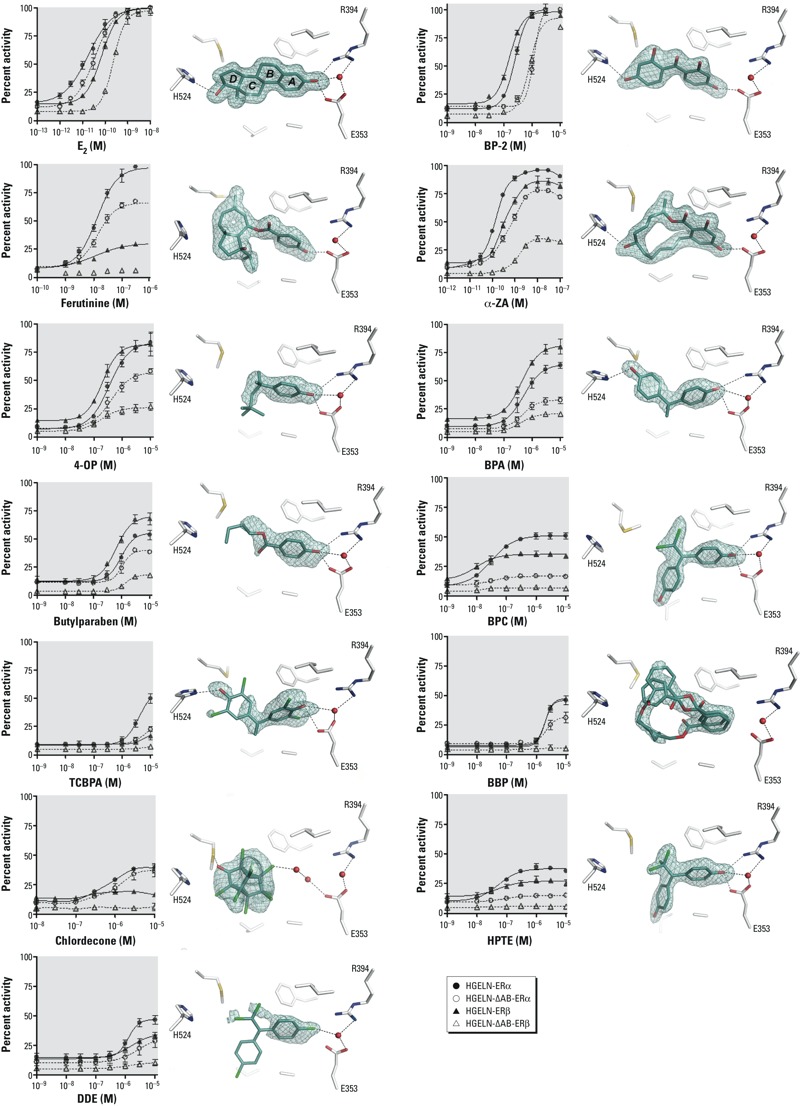
The relative activity of xenoestrogens relies on their different binding modes. (Left) Dose–response curves corresponding to the HGELN-ERα, -∆AB-ERα, -ERβ, and -∆AB-ERβ, luciferase assays of E_2_ and xenoestrogens. The maximal activity (100%) was obtained with 10 nM E_2_; values are mean ± SD from three separate experiments. (Right) The interaction networks of E_2_ and xenoestrogens with LBD residues of ERα. Key for structures: red, oxygen; blue, nitrogen; cyan, carbon; yellow, sulfur; green, chlorine; black dashed lines, hydrogen bonds; red spheres, water molecules. The electron density represents a *F_o_–F_c_* simulated annealing omit map contoured at 3σ.

**Figure 3:**
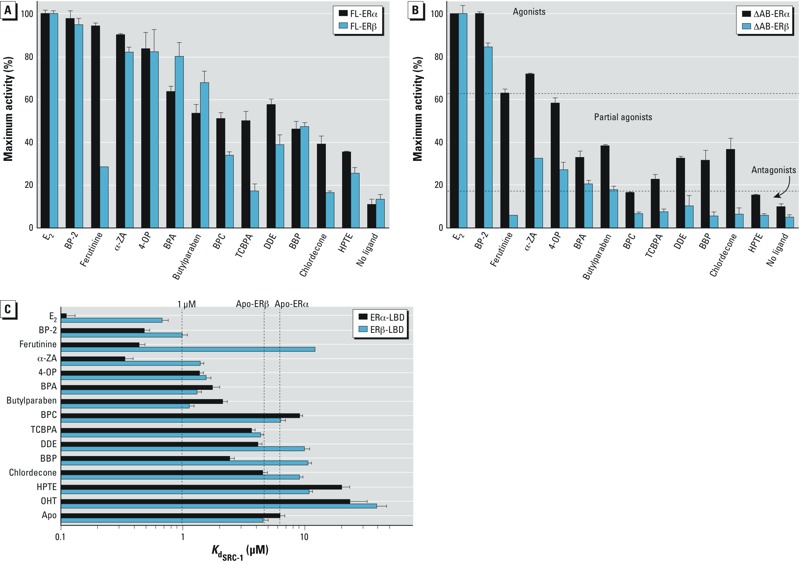
Differential involvement of AFs in ERs. HGELN-ERs cells (*A*) and HGELN-∆AB-ERs cells (*B*) were incubated with 10 nM E_2_ or 10 μM ER exogenous ligands. The maximal luciferase activity (100%) was obtained with 10 nM E_2_; values are the mean ± SD from three separate experiments. (In *B*, the horizontal dotted lines highlight the partition of the ligands into three classes based on fluorescence anisotropy data. (*C*) Fluorescence anisotropy data showing the relative affinity of the SRC-1 NR2 peptide for ERα LBD or ERβ LBD in the absence of ligand or in the presence of saturating concentrations of E_2_ or xenoestrogens. Ligands are classified as agonists (*K*_d_ ≤ 1 μM), partial agonists (1 μM ≤ *K*_d_ ≤ values for apo-ERα or -ERβ), or antagonists (*K*_d_ ≥ values for apo-ERα or -ERβ).

*Compound activities on ER*α *and ER*β. We monitored the agonistic potential of the compounds using stably transfected HGELN-ERα and -ERβ cell lines, allowing for a comparison of the effect of compounds on both human ER subtypes in a similar cellular context. All compounds were first tested on the HGELN parental cell line containing only the reporter gene. We observed some cytotoxicity at ligand concentrations of ≥ 10 μM but no unspecific modulation of luciferase expression (data not shown). We then characterized the activity of the compounds on HGELN-ER cell lines containing full-length (FL) ERα or ERβ. As shown in Figures 2 and 3A, the agonistic potentials depend on the receptor subtype and vary drastically among molecules that range from full agonists to weak agonists or antagonists. Whereas BP-2 acted as a full agonist of both ER subtypes, ferutinine was a selective activator of ERα, and α-ZA efficiently activated both receptors with a slight preference for ERα. The remainder of the compounds can be considered partial agonists with graded effects, with 4-OP being the most active (~ 80% of the transactivation seen with E_2_) and TCBPA inducing only 17% activity in the HGELN-ERβ cell line. Interestingly, some of these compounds displayed different activation capabilities of the two receptor subtypes, as illustrated by TCBPA and chlordecone, which were significantly more efficacious for ERα (50% and 39%, respectively) than for ERβ (17% and 19%, respectively). In total, three compounds activated the two ER subtypes equally (BP-2, 4-OP, and BBP), two compounds activated ERβ more efficiently than ERα (BPA and butylparaben), and seven compounds activated ERα more efficiently than ERβ (ferutinine, α-ZA, BPC, TCBPA, DDE, chlordecone, and HPTE). The EC_50_ (median effective concentration) values derived from the transactivation curves suggest that the compounds bound to both ER subtypes with similar affinities (see Supplemental Material, Table S1). To validate this observation, we performed competitive binding assays with (^3^H)-E_2_ (see Supplemental Material, Figure S1 and Table S2), which showed a wide array of affinities ranging from subnanomolar to micromolar values. Together, these experiments show that, in the context of HeLa cells, all the molecules bound to FL-ERα and FL-ERβ without subtype selectivity, whereas the functional outcomes of these interactions were, in most cases, subtype specific.

*Differential usage of AF-1 and AF-2*. Having characterized the estrogenic potential of the compounds on FL-ERα and FL-ERβ, we performed additional cell-based experiments aimed at assessing the relative contribution of the ERs’ AF-1 and AF-2 to this activity. We examined the agonistic properties of compounds using HELN cells stably transfected with ERs that had their N-terminal AB (AF-1) region deleted (ΔAB-ERα and ΔAB-ERβ). Interestingly, we observed that deletion of the AB domains reduced the xenoestrogen-induced transcriptional activity of ERs, with the effect being remarkably more pronounced for ERβ (Figure 2, and compare Figure 3A and 3B). Correlation diagrams representing the maximum activity of FL-ERs versus ΔAB-ERs in the presence of the various ligands clearly illustrate the differential involvement of the two AFs in ERα and ERβ (see Supplemental Material, Figure S2). Among the 12 compounds tested, only 2 displayed a fully AF-1–independent activity. These 2 compounds are BP-2, which acted as a full agonist of the entire or truncated ER forms, and more surprisingly, chlordecone, whose partial agonist activity on ERα also relied exclusively on the AF-2. Comparing the decrease in the agonistic potential of the other compounds upon AF-1 removal in ERα and ERβ, we observed that most of them retained a significant degree of activity in HGELN-ΔAB-ERα but not in HGELN-ΔAB-ERβ cell lines, where the majority of the ER ligands become partially or completely inactive. Together, these data suggest that, in the context of HeLa cells, the environmental compounds bind to ERα and ERβ with similar affinities but modulate the transcriptional activity of the two subtypes through different combinations of AF-1 and AF-2, with AF-1 being preeminent in the EDC-induced transcriptional activity of ERβ.

*Subtype-specific AF-2 modulation by exogenous ER ligands*. To evaluate whether the differential involvement of the N- and C-terminal AFs is dependent on the cellular context or a consequence of intrinsic differences between the two ER subtypes, we used fluorescence anisotropy to monitor the recruitment of a fluorescein-labeled peptide derived from the coactivator SRC-1 by the purified ERα- or ERβ-LBDs in either the absence (apo-receptor) or the presence of compounds. This experimental setup allows measuring the influence of a given ligand on a receptor’s AF-2 in purely *in vitro* conditions. In keeping with their agonistic or antagonistic activities, E_2_ and OHT, respectively, strongly enhanced and decreased the binding affinity of SRC-1 to both ERs, with E_2_ being slightly more efficient on ERα (Figure 3C; see also Supplemental Material, Figure S3 and Table S3). The values of the dissociation constants (*K*_d_) obtained in the presence of the various compounds show that based on their impact on the recruitment of the coactivator-derived peptide by ERs, the ligands can be partitioned into three classes. The first class corresponds to strong agonists (*K*_d_ ≤ 1 μM) and includes α-ZA, ferutinine, and BP-2 for ERα but only BP-2 for ERβ. The second class comprises the partial agonists (1 μM ≤ *K*_d_ ≤ values for apo-ERα or -ERβ) and contains most of the remaining molecules for ERα (4-OP, BPA, butylparaben, BBP, TCBPA, chlordecone, and DDE), but only α-ZA, 4-OP, BPA, and butylparaben for ERβ. Finally, the third class encompasses the antagonists (*K*_d_ ≥ values for apo-ERα or -ERβ): BPC and HPTE for ERα, and ferutinine, BBP, BPC, TCBPA, chlordecone, HPTE, and DDE for ERβ. These fluorescence anisotropy data are in good agreement with the transactivation assays because all of the activity profiles measured in HGELN-ΔAB-ER cell lines (Figure 3B) fell into these three categories of ligands, confirming that the compounds activated ERα AF-2 more efficiently than ERβ AF-2, which appears more prone to antagonism. Thus, binding of environmental ligands imposed different structural constraints on the LBDs of the two ER subtypes, which most likely account for the differential contribution of both AFs in ERα and ERβ.

*Structural analysis*. To gain structural insights into the mechanisms by which exogenous ligands bind to and activate ERs, we solved the crystal structures of ERα LBD in complex with the various compounds. Because ERα- and ERβ-LBDs share a high degree of homology in their amino acid sequence and are very similar in their ternary architecture, we reasoned that structural information regarding interaction with ERβ could be acquired through molecular modeling using the ERα LBD structures. To allow the crystallization of ERα bound to the environmental molecules acting essentially as partial agonists that are unable to induce a stable conformation of the LBD, we used the ERα-Y537S LBD mutant described previously by Nettles et al. (2008). In several independent studies, the Y537S surface mutation has been shown to stabilize the active conformation of the receptor, and, in turn, facilitate crystallization of weak agonists without modifying either the overall architecture of the LBD or the binding mode of ligands (Bruning et al. 2010; Delfosse et al. 2012; Nettles et al. 2008). To further stabilize the ERα-Y537S LBD in its active form, a peptide containing the second interaction motif of the coactivator SRC-1 was also added during the crystallization trials. Details of the structure determination and refinement are summarized in Supplemental Material, Table S4.

**Figure 4:**
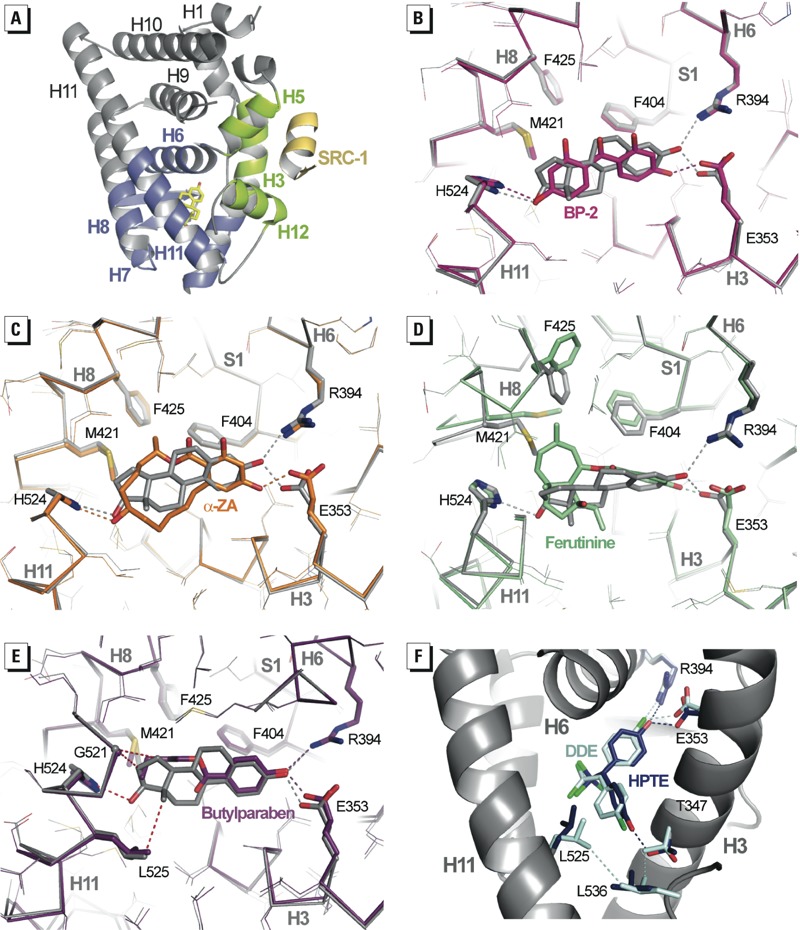
Xenoestrogens use diverse binding modes. (*A*) The entire structure of the ERα LBD in complex with E_2_ and SRC-1 coactivator peptide (yellow); the structure shows the AF‑2 surface formed by helices H3, H5, and H12 (green), and the lower part of the LBD (blue) encloses the ligand-binding pocket (LBP). (*B–E*) Interaction networks of BP-2 (*B*; pink), α-ZA (*C*; orange), ferutinine (*D*; green), and butylparaben (*E*; purple) with residues of the LBP compared with that of E_2_ (gray). In (*E*), red dashed lines represent the interactions lost in the butylparaben complex structure. (*F*) HPTE and DDE adopt the orientation previously observed for BPC allowing HPTE to interact with residue T347. This position results in the disruption of the hydrophobic network involving helices H3 and H11 and the loop preceding H12, thereby destabilizing the AF‑2 surface. Color code: red, oxygen; blue, nitrogen; cyan, carbon; yellow, sulfur; green, chlorine; dashed lines, hydrogen bonds and hydrophobic interactions.

The structures display the canonical active conformation, with helix H12 capping the ligand-binding pocket (LBP) and the SRC-1 peptide bound to the “AF-2 surface” formed by helices H3, H5, and H12 (Figure 4A). Most compounds could be precisely placed in their respective electron density, revealing different binding modes (Figure 2). Some ligands, such as BP-2, α-ZA, BPA, and TCBPA, adopted a binding mode reminiscent of that used by E_2_, with two phenol groups hydrogen-bonded to three polar residues located at the two ends of the LBP, namely H524 (H11) on one side and E353 (H3) and R394 (H6) on the other side. However, we also noticed significant differences in the geometry of the interactions between H524, E353, R394, and the hydroxyl moieties of E_2_ and the ligands with possible functional and/or binding implications. Indeed, none of the compounds recapitulated the exact hydrogen bond network seen in the E_2_-containing complex. The remaining contacts involved essentially van der Waals interactions, the number of which varies from one compound to another and accounts in part for the various binding affinities of the ligands. Several compounds did not interact with H524 because they lack a second hydroxyl group (ferutinine, 4-OP, butylparaben, and DDE) or because they adopt a position that draws this hydroxyl moiety toward T347 in H3 (BPC, HPTE, and DDE). Finally, two compounds, BBP and chlordecone, were not engaged in any direct interaction with either of these polar residues, the latter being indirectly hydrogen bonded to E353 via water molecules. As shown in Figure 2, the position of DDE could not be precisely determined because of the absence of electron density for some regions of the ligand. This poorly defined electron density reflects a higher dynamic for DDE. Finally, the docking of two BBP molecules with distinct positions was necessary to fully account for the observed electron density, indicating that this molecule can adopt two alternate orientations in the LBP.

*Structural basis for compound actions in ER*α. We next considered how these different binding modes may account for the various activity profiles of compounds toward ERα. Superposition of our 13 structures onto the structure in complex with E_2_ revealed that the regions of the LBP occupied by the ligand vary greatly from one compound to another. BP-2, which displayed the highest agonistic activity among the molecules used in this study, occupies almost the same volume as E_2_ in the LBP and therefore imposes similar side chain conformations (Figure 4B). In particular, it stabilizes the same conformer of H524 as that seen in the E_2_-bound ERα. We and others have previously shown that this residue is involved in a key hydrogen bond network including residues from H3 and H11, which are part of the docking site maintaining H12 in the active position (Bruning et al. 2010; Delfosse et al. 2012; Nettles et al. 2008). α-ZA occupies a bit more space than E_2_, especially in the H8 region on one side and the H3, H11, and H12 region on the other side (Figure 4C). Interestingly, ERα accommodates this larger compound with only minimal LBP rearrangements. In contrast, a number of large side chain conformational changes are necessary to accommodate the ferutinine. In particular the bulky methyl-cycloheptene ring pointing toward H8 provokes the enlargement of a small preexisting hydrophobic cavity via the reorientations of M421 and F425 in H8, as well as F404 in the β-strand S1 (Figure 4D). This structure further highlights the plasticity of this particular region of ERα LBP (Nettles et al. 2007), which can accommodate bulky ligands within the confined environment of the active conformation while retaining a substantial level of agonistic activity (Figure 2).

In contrast with the aforementioned compounds, ligands with partial AF-2 agonist activity are generally smaller in size (4-OP, BPA, butylparaben, chlordecone) and/or are more flexible (4-OP, butylparaben, BBP). As a consequence, they are less efficient in stabilizing the LBP side chain conformations required to hold the active form of the receptor. The loss of stabilizing contacts triggered by bisphenols and their functional outcome has previously been discussed in detail (Delfosse et al. 2012). The suboptimal interaction between ERα and the weak compounds reported in this study is exemplified by the structure of the butylparaben-containing complex, where several previously recognized ligand-H11 stabilizing interactions are missing (Figure 4E). We have previously shown that the two chlorine atoms of BPC prevent this compound from adopting the same position as BPA in the ERα LBP (Figure 2) and that the AF-2 antagonistic character of BPC is a direct consequence of this particular binding mode. Indeed, the phenol ring, which adopts a different orientation in BPC, induces a 180° rotation of T347 (H3) and forms a hydrogen bond with the hydroxyl group of this residue. The complete reorientation of the T347 side chain provokes the disruption of a cluster of hydrophobic interactions, which hold together H3, H11, and the loop preceding H12, thereby destabilizing the AF-2 surface. It is noteworthy that the closely related HPTE adopts a similar binding mode and acts as an AF-2 antagonist, whereas DDE, which contains chlorine atoms instead of hydroxyl groups, does not induce the reorientation of T347 and acts as a partial agonist (Figure 4F). Together, these experimental data provide a structural rationale to explain the functional properties of the environmental ligands of ERs at a near-atomic level.

*Structural basis for subtype-specific action of ER ligands*. We subsequently looked for a rational explanation for the differential activation of ERα and ERβ AF-2 by the compounds (Figure 3B). The LBDs of the two ER subtypes share a high degree of homology in their primary sequence. Notably, there are only two conservative residue substitutions in the LBPs of the two receptors. These substitutions correspond to the replacement of L384 (ERα) by M336 (ERβ) in H6 and M421 (ERα) by I373 (ERβ) in H8, which have been shown previously to account, at least in part, for the subtype-specific action of ER ligands (Nettles et al. 2004). Inspection of the crystal structures of both ER subtypes in complex with E_2_ shows that the variable amino acids reside on each side of the C and D rings of E_2_ and create different space constraints in this portion of ERα and ERβ LBPs (see Supplemental Material, Figure S4). Indeed, superposition of our ERα or ERβ structures contained in the Protein Data Bank (PDB) reveals that in ERα, M421 (H8) can adopt a wide array of conformations to accommodate the ligands, whereas in ERβ, M336 (H6) is much less flexible due to strong steric constraints provided by surrounding residues (see Supplemental Material, Figure S4). Thus, with two bulky and rigid residues in H6 (M336) and H8 (I373), ERβ might be more sensitive than ERα to variations in the size of the bound ligand.

**Figure 5:**
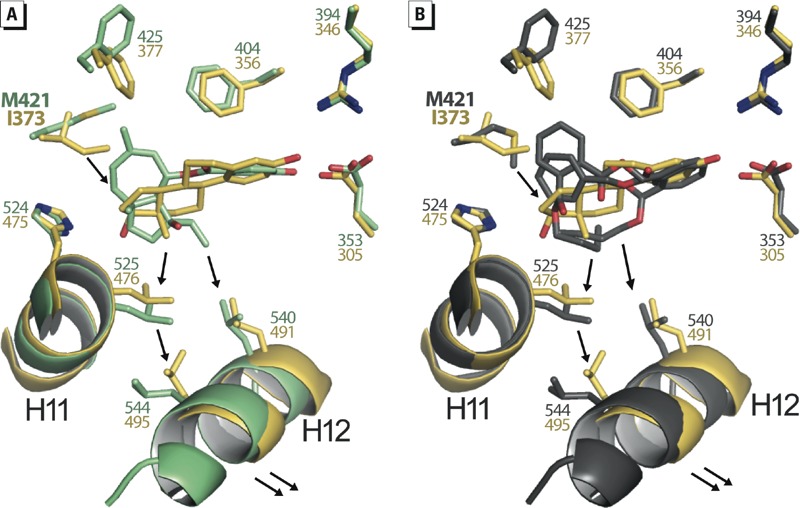
Methionine 421 confers plasticity and adaptability to ERα LBP. Structure superposition of E_2_-bound ERβ LBD (yellow) with (*A*) ferutinine-bound ERα LBD (green), or (*B*) BBP-bound ERα LBD (gray). The presence of I373 in ERβ instead of M421 in ERα will induce a shift of bulky ligands toward helix H12 thus lowering the stability of the AF‑2.

Accordingly, our structures reveal that most of the compounds insert a bulky feature in this region of the LBP and exhibit a marked subtype-dependent activity as illustrated by ferutinine and BBP (Figures 5 and 3B). Both molecules contain a bulky group that projects toward ERα M421, which, in turn, must undergo a large conformational change. In ERβ, the linear amino acid M421 is replaced by the branched residue I373, which is unable to move away from the pocket and make room for the ligands. A likely consequence is that I373 induces a shift of the ligands toward H12, thus lowering the interaction of H12 in the active conformation with the LBD surface and accounting for the weakest agonistic activity of the compounds in ERβ (Figure 5). Obviously the strength of the steric constraints applied to ERβ H12 varies according to the chemical structure of the bound ligand, as reflected by the graded partial agonistic/antagonistic activity of the compounds (Figure 3B). Finally, it is noteworthy that the weak ligand-induced ERβ AF-2 activity can be partially or completely compensated by the N-terminal activation domain, thus confirming the preeminent functional role of ERβ AF-1 in HeLa cells (Figure 3A,B).

## Discussion

Deregulation of NR-mediated transcription accounts for the deleterious effects of many EDCs. Thus, characterization of the interaction between receptors and environmental compounds both at the structural and functional levels, as well as the development of robust *in vivo*, *in vitro*, and *in silico* screening methods, are important for the assessment of the global hormonal activity of a large number of chemicals (Janosek et al. 2006). In this context, we have used a combination of complementary biochemical, structural, biophysical, and cell-based approaches to provide a mechanistic view for how members of the most recognized pollutant families bind to and activate ERα and ERβ, two of their primary biological targets.

We observed that the compounds bind to both ER subtypes with similar affinities but modulate the transcriptional activity of ERα and ERβ in different manners. Using HeLa cells stably transfected with ΔAB-ERs and fluorescence anisotropy measurements with purified ER LBDs, we showed that the environmental compounds activated the C-terminal AF-2 of ERα more efficiently than that of ERβ. However, when the N-terminal AF-1 was present (FL-ERs), the global activity of ERβ was partially (α-ZA, BPC, DDE, HPTE) or completely (BP-2, 4-OP, BBP) restored compared with the results obtained with ERα, in which two ligands (BPA and butylparaben) activated FL-ERβ more efficiently than FL-ERα. In contrast, ferutinine, TCBPA, and chlordecone acted as ERβ antagonists in both the FL and ΔAB constructs. These data clearly show that the environmental ligands acted in a subtype-specific fashion as full agonists, partial agonists, or antagonists by using different combinations of the N- and C-terminal activation functions of ERα and ERβ, the AF-1 being dominant in the latter. They also suggest that the binding of structurally diverse molecules induced specific ER LBD structures and/or dynamics with divergent impacts on AF-1 activity (compare BBP and chlordecone on ERβ in Figure 3A,B). However, the precise structural basis of this interdomain communication is still unknown because no three-dimensional (3D) structure of an entire NR has yet been obtained.

The relative agonist/antagonist activity of a given ER ligand is dependent on several parameters, including the nature of the target gene and cell type considered, the latter being critical due to variation in the equipment of transcription coregulators that finely tune the activity of the two AFs of the ERs (McDonnell et al. 2002). Such considerations might explain, for instance, why HPTE is characterized here as an antagonist but reported to display agonistic properties in other studies (Wilson et al. 2004). In a similar manner, Li et al. (2013) observed that chlordecone activated ERα in HeLa cells, but it was unable to do so in HepG2 cells. It would be therefore interesting to extend the present study by comparing the effects of the various ER ligands on ER activities in different cell types and on the recruitment of several coregulatory proteins. In terms of biological activity, environmental estrogens are comparable to the selective estrogen receptor modulators (SERMs) such as raloxifen and tamoxifen (which also act as partial agonists) and selectively block estrogen action in the breast but not in other tissues, such as bones (Nilsson and Gustafsson 2011). As observed for these SERMs, it appears quite obvious that xenoestrogens presenting partial agonism will manifest different activity depending on the tissues. In line with this, further work will be needed to define the deleterious effects linked to the selective partial agonistic activity of environmental chemicals on key physiological processes regulated by ERs such as bone and metabolic homeostasis, vasomotor symptoms, depression, or neurodegenerative diseases (Nilsson and Gustafsson 2011).

It is well established that ERα is the major driver of the proliferative effects of estrogens in cancers of both breast and ovary, as well as in normal reproductive tissues, with ERβ serving largely as a brake for ERα-driven proliferation (Bossard et al. 2012; Madak-Erdogan et al. 2013; Paruthiyil et al. 2004; Strom et al. 2004). Indeed, in ovarian cancer BG1 cells expressing both ERs, we previously observed that ERα-selective agonists activated cell proliferation more efficiently than did ERβ-selective ligands or ER pan-agonists (Docquier et al. 2013). This observation, together with our finding that ER environmental ligands may act in a subtype-specific fashion, suggests that different xenoestrogens could have different impacts on cancer incidence. In this regard, one can predict that pollutants acting as ERα agonists and ERβ antagonists in a particular cellular context (e.g., ferutinine in HeLa cells) should stimulate cell proliferation and tumor growth more effectively than compounds activating more ERβ than ERα.

Finally, our crystallographic analysis revealed the various mechanisms by which distantly related chemicals bind to and activate ERs. These data will increase the effectiveness of 3D structure-based computational tools aimed at predicting the NR-mediated activity of environmental pollutants (Delfosse et al. 2012; Vuorinen et al. 2013). In addition to providing a better understanding of the differential activities, binding affinities, and specificities of environmental ER ligands, the structures provide rational guidelines for the design of safer chemicals.

## Supplemental Material

(899 KB) PDF
