# Supplementary material for: Structural and Functional Profiling of Environmental Ligands for Estrogen Receptors
Source: Environ Health Perspect. 2014 Sep 26;122(12):1306–13. doi: 10.1289/ehp.1408453 (PMC4256047; doi:10.1289/ehp.1408453)
Supplement: (899 KB) PDF [file ehp.1408453.s001.508.pdf]

Environmental Health Perspectives doi:10.1289/ehp.1408453

Supplemental Material

Structural and Functional Profiling of Environmental Ligands for Estrogen Receptors

Vanessa Delfosse, Marina Grimaldi, Vincent Cavaillès, Patrick Balaguer, and William Bourguet

Methods

Reporter cell lines and culture conditions

The stably transfected luciferase reporter HELN-ERα, -ERβ, -ΔAB-ERα and -ΔAB-ERβ cell lines have been already described (Molina-Molina et al. 2008). Briefly, generation of these cells was performed in two steps. The estrogen responsive reporter gene was first stably transfected into HeLa cells, generating HGELN cell line (named HELN for HeLa-GAL_4RE-ERE-Luciferase-Neo) and, in a second step, these HELN cells were transfected with human ERα, ERβ, -ΔAB-ERα and -ΔAB-ERβ plasmid constructs to obtain the HELN-ERα,-ERβ, -ΔAB-ERα and -ΔAB-ERβ cell lines, respectively. Luciferase, cell proliferation and whole-cell ER competitive binding assays have been performed as described in (Molina-Molina et al. 2008). Tissue culture plates used in this study came from Greiner Bio-one (Monroe, NC, USA), and media was purchased from Invitrogen (Grand Island, NY, USA). Luciferin (sodium salt) was purchased from Promega (Charbonnières, France).

Protein production and purification

The human wt-ERα LBD and the ERα-Y537S LBD mutant (amino acids 302-552) were cloned into a modified pET-15b vector resulting in a fusion protein containing a thioredoxine-hexahistidine tag at the N-terminus, and purified as described in (Delfosse et al. 2012). The human wt-ERβ LBD (amino acids 261-502) was cloned into a pET-32a vector. Gene expression was induced in BL21(DE3) cells overnight at 18°C in LB medium without any ligand and the protein was purified in the apo form. The cell lysate was first applied onto a nickel affinity column (HiTrap 5 mL; GE Healthcare). The eluted protein was then applied onto a desalting column (HiPrep 26/10 Desalting; GE Healthcare) equilibrated with 20 mM Tris pH 8.0, 50 mM NaCl, 5 mM DTT and 5% glycerol and the N-terminal thioredoxine-hexahistidine tag was subsequently removed by adding TEV protease overnight at 4°C. The protein was separated from the cut tag by size exclusion chromatography (Superdex 75 HR 16/60; GE Healthcare) and then concentrated in the gel filtration buffer (20 mM Tris pH 8.0, 150 mM NaCl, 5 mM DTT and 10% glycerol). Proteins are stored at -40°C.

Data collection and structure determination

For all complexes, native data were collected from one crystal cryoprotected with 30% glycerol on the ID23-1, ID23-2 or ID29 beamlines at the European Synchrotron Radiation Facility, Grenoble, France. Data were processed and scaled with XDS and XSCALE (Kabsch 2010). Crystals belong to space group $P2_1$ for all complexes. The X-ray structures were solved by

molecular replacement method using PHENIX (phenix.automr) (Adams et al. 2010), and refinement and rebuilding were performed with COOT (Emsley and Cowtan 2004), PHENIX (phenix.refine) (Adams et al. 2010) and REFMAC (Murshudov et al. 1997) from the CCP4 (Winn et al. 2011) suite. Data collection and refinement statistics are summarized in Supplemental Material, Table S4. Figures were prepared with PyMOL (http://pymol.org/).

References

Adams PD, Afonine PV, Bunkoczi G, Chen VB, Davis W, Echols N, et al. 2010. Phenix: A comprehensive python-based system for macromolecular structure solution. Acta Crystallogr D Biol Crystallogr 66:213-221.

Delfosse V, Grimaldi M, Pons JL, Boulahtouf A, le Maire A, Cavailles V, et al. 2012. Structural and mechanistic insights into bisphenols action provide guidelines for risk assessment and discovery of bisphenol a substitutes. Proc Natl Acad Sci U S A 109:14930-14935.

Emsley P, Cowtan K. 2004. Coot: Model-building tools for molecular graphics. Acta Crystallogr D Biol Crystallogr 60:2126-2132.

Kabsch W. 2010. Xds. Acta Crystallogr D Biol Crystallogr 66:125-132.

Molina-Molina JM, Escande A, Pillon A, Gomez E, Pakdel F, Cavaillès V, et al. 2008. Profiling of benzophenone derivatives using fish and human estrogen receptor-specific in vitro bioassays. Toxicology and Applied Pharmacology 232(3):384-395.

Murshudov GN, Vagin AA, Dodson EJ. 1997. Refinement of macromolecular structures by the maximum-likelihood method. Acta Crystallogr D Biol Crystallogr 53:240-255.

Winn MD, Ballard CC, Cowtan KD, Dodson EJ, Emsley P, Evans PR, et al. 2011. Overview of the ccp4 suite and current developments. Acta Crystallogr D Biol Crystallogr 67:235-242.

Table S1. EC_{50} values derived from the dose-response curves in Figure 2. Values are the mean ± SD from three separate experiments.

**Ligand**	**ERα (M)**	**ΔAB-ERα (M)**	**ERβ (M)**	**ΔAB-ERβ (M)**
E_2	$1.77 \pm 0.66\ 10^{-11}$	$3.47 \pm 0.70\ 10^{-11}$	$6.95 \pm 0.88\ 10^{-11}$	$2.34 \pm 0.22\ 10^{-10}$
BP-2	$2.54 \pm 0.35\ 10^{-7}$	$1.16 \pm 0.03\ 10^{-6}$	$1.53 \pm 0.42\ 10^{-7}$	$1.35 \pm 0.05\ 10^{-6}$
Ferutinine	$1.30 \pm 0.37\ 10^{-8}$	$1.35 \pm 0.70\ 10^{-8}$	$9.12 \pm 4.50\ 10^{-9}$	ND
α-ZA	$1.38 \pm 0.25\ 10^{-10}$	$4.84 \pm 1.96\ 10^{-10}$	$3.49 \pm 0.69\ 10^{-10}$	$1.28 \pm 0.31\ 10^{-9}$
4-OP	$3.48 \pm 1.24\ 10^{-7}$	$4.11 \pm 2.29\ 10^{-7}$	$1.97 \pm 0.16\ 10^{-7}$	$1.82 \pm 0.93\ 10^{-7}$
BPA	$5.09 \pm 0.95\ 10^{-7}$	$4.66 \pm 0.22\ 10^{-7}$	$4.24 \pm 0.29\ 10^{-7}$	$3.92 \pm 0.75\ 10^{-7}$
Butylparaben	$9.54 \pm 0.29\ 10^{-7}$	$1.04 \pm 0.45\ 10^{-6}$	$6.31 \pm 2.31\ 10^{-7}$	$1.34 \pm 0.44\ 10^{-6}$
BPC	$3.09 \pm 0.29\ 10^{-8}$	$3.34 \pm 1.14\ 10^{-8}$	$1.03 \pm 0.36\ 10^{-8}$	ND
TCBPA	$1.15 \pm 0.19\ 10^{-5}$	ND	ND	ND
DDE	$9.00 \pm 4.94\ 10^{-7}$	$5.57 \pm 2.38\ 10^{-6}$	$2.13 \pm 0.67\ 10^{-6}$	ND
BBP	$1.93 \pm 0.08\ 10^{-6}$	$2.13 \pm 0.34\ 10^{-6}$	$1.94 \pm 0.05\ 10^{-6}$	ND
Chlordecone	$6.57 \pm 2.45\ 10^{-7}$	$1.09 \pm 0.56\ 10^{-6}$	ND	ND
HPTE	$5.30 \pm 2.40\ 10^{-8}$	$8.33 \pm 3.00\ 10^{-8}$	$5.22 \pm 3.24\ 10^{-8}$	ND

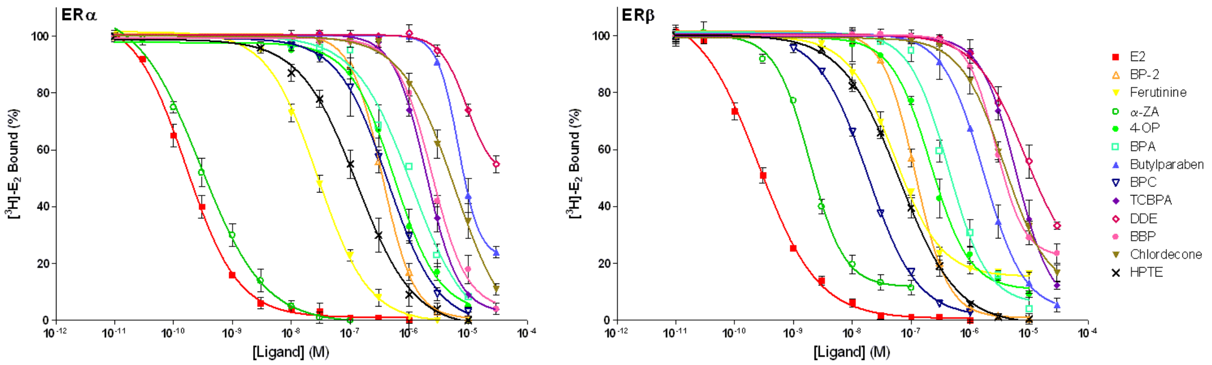

Figure S1. Competition inhibition of [^{3}H]-E_2 binding to ERα and ERβ by xenoestrogens. HELN-ERα and -ERβ cells were incubated with different concentrations (10 pM - 30 μM) of E_2 and xenoestrogens in the presence of 0.1 nM [^{3}H]-E_2. Values are the mean ± SD from three separate experiments.

Table S2. E_2 and xenoestrogens K_d values derived from curves shown in Supplemental Material, Figure S1. Values are the mean ± SD from three separate experiments.

**Ligand**	**ERα (M)**	**ERβ (M)**
E_2	1.63 ± 0.31 10^{-10}	2.55 ± 0.93 10^{-10}
BP-2	3.52 ± 0.32 10^{-7}	1.17 ± 0.11 10^{-7}
Ferutinine	2.62 ± 0.46 10^{-8}	5.07 ± 1.07 10^{-8}
α-ZA	2.88 ± 1.51 10^{-10}	1.88 ± 0.30 10^{-9}
4-OP	5.68 ± 1.95 10^{-7}	2.11 ± 0.54 10^{-7}
BPA	9.84 ± 0.34 10^{-7}	5.25 ± 1.77 10^{-7}
Butylparaben	6.73 ± 0.28 10^{-6}	1.68 ± 0.08 10^{-6}
BPC	4.22 ± 0.33 10^{-8}	1.99 ± 0.11 10^{-8}
TCBPA	1.91 ± 0.21 10^{-6}	6.04 ± 0.72 10^{-6}
DDE	9.24 ± 1.10 10^{-6}	8.40 ± 3.90 10^{-6}
BBP	2.45 ± 0.57 10^{-6}	2.70 ± 0.10 10^{-6}
Chlordecone	5.77 ± 2.41 10^{-6}	3.74 ± 0.90 10^{-6}
HPTE	1.27 ± 0.34 10^{-7}	5.98 ± 0.85 10^{-8}

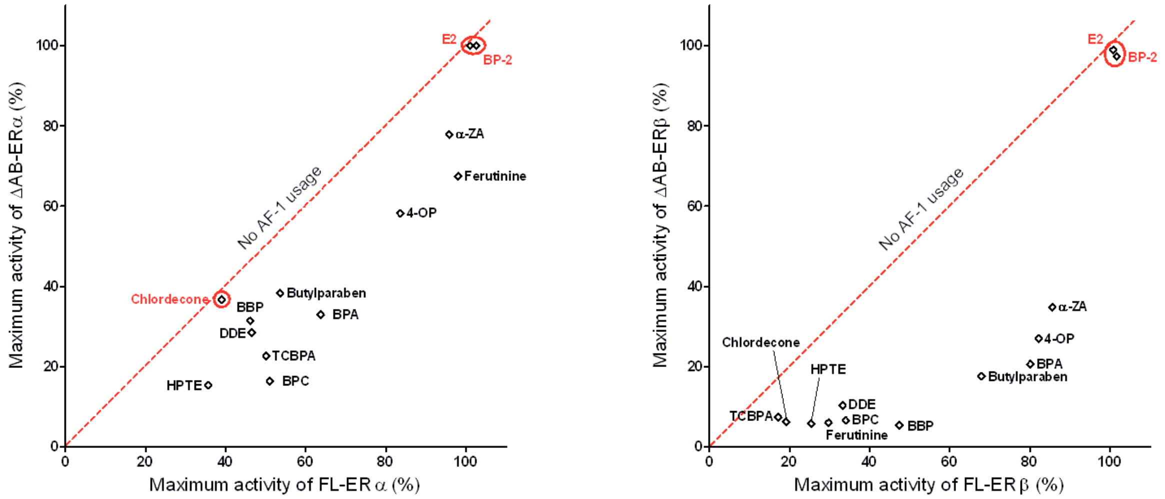

Figure S2. Correlation diagrams representing the maximum activity of FL-ERs *vs* ΔAB-ERs in the presence of E_2 and the different xenoestrogens in HeLa cells.

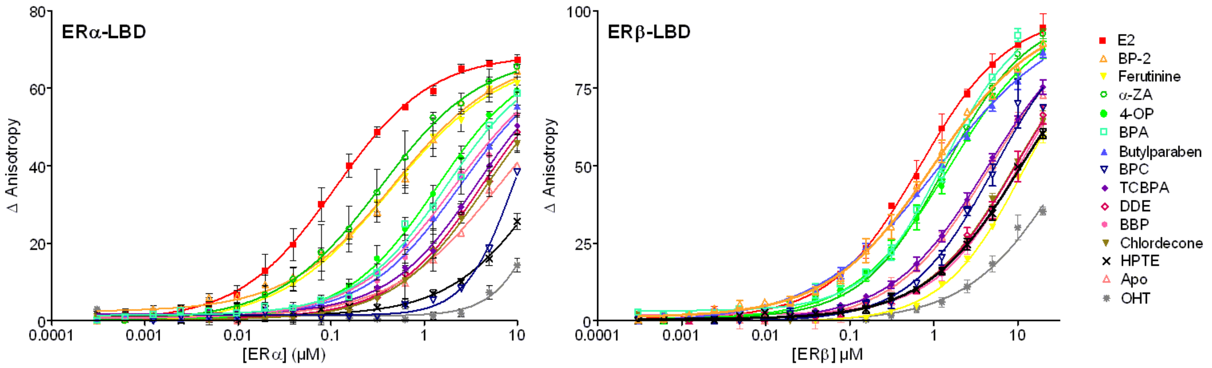

Figure S3. Titration of fluorescein-labeled SRC-1 NR2 peptide by wt-ERα LBD and wt-ERβ LBD in the absence of ligand or in the presence of E_2, OHT or the different xenoestrogens.

Table S3. SRC-1 NR2 peptide K_d values derived from curves shown in Supplemental Material, Figure S3. Values are the mean ± SD from three separate experiments.

**Ligand**	**ERα-LBD (μM)**	**ERβ-LBD (μM)**
E_2	0.11 ± 0.02	0.68 ± 0.08
BP-2	0.48 ± 0.06	0.99 ± 0.11
Ferutinine	0.44 ± 0.05	12.11 ± 0.55
α-ZA	0.33 ± 0.06	1.39 ± 0.09
4-OP	1.37 ± 0.11	1.55 ± 0.15
BPA	1.76 ± 0.25	1.31 ± 0.11
Butylparaben	2.11 ± 0.20	1.13 ± 0.11
BPC	9.07 ± 0.51	6.32 ± 0.65
TCBPA	3.67 ± 0.24	4.33 ± 0.27
DDE	4.10 ± 0.36	9.93 ± 1.10
BBP	2.42 ± 0.24	10.61 ± 0.73
Chlordecone	4.53 ± 0.44	9.13 ± 0.58
HPTE	20.19 ± 3.37	10.91 ± 0.70
Apo	6.28 ± 0.63	4.54 ± 0.46
OHT	23.63 ± 8.97	38.76 ± 8.15

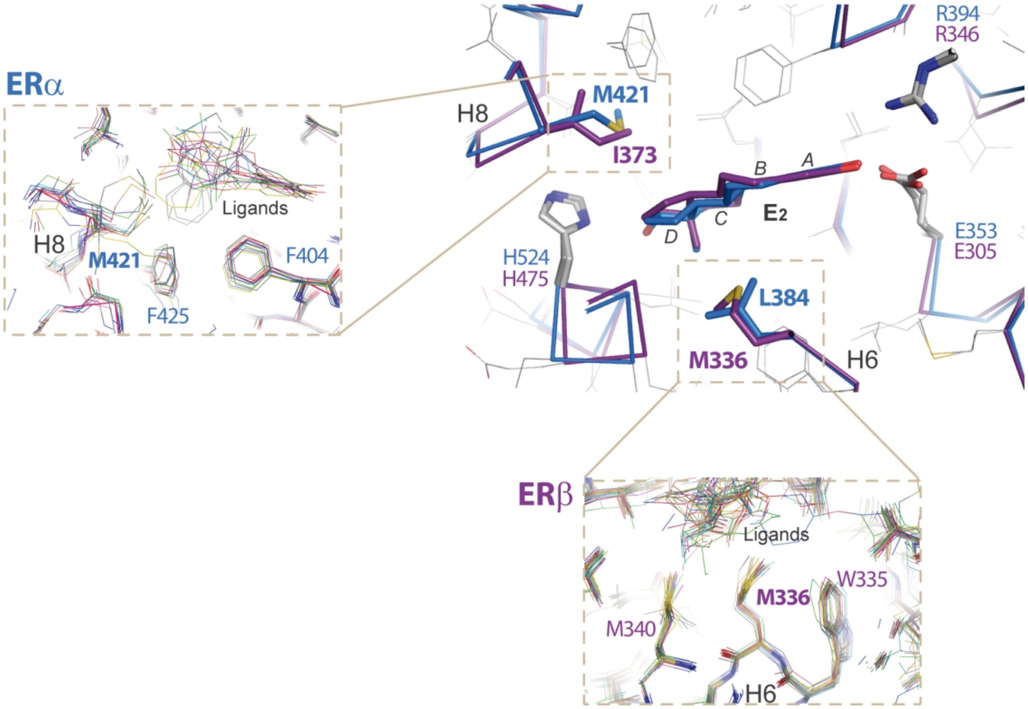

Figure S4. ERα and ERβ ligand-binding pocket (LBP) methionines flexibility. Superposition of E_2-bound ERα (in blue) and E_2-bound ERβ (in purple) showing the two specific positions of their LBPs. Boxes show the relative flexibility of ERα-M421 (superposition of our 12 EDC/ERα structures) and ERβ-M336 (superposition of the 34 ERβ structures contained in the PDB).

Table S4. Data collection and refinement statistics.

Variable	Chlordecone (4MG5)	BBP (4MG6)	Ferutinine (4MG7)	α-ZA (4MG8)	Butylparaben (4MG9)	4-OP (4MGA)	TCBPA (4MGB)	BP-2 (4MGC)	HPTE (4MGD)
**Data collection**									
Space group	$P\,2_1$	$P\,2_1$	$P\,2_1$	$P\,2_1$	$P\,2_1$	$P\,2_1$	$P\,2_1$	$P\,2_1$	$P\,2_1$
Cell dimensions									
a, Å	54.70	54.48	54.55	55.95	54.92	54.88	54.67	56.02	54.90
b, Å	81.78	81.24	81.62	81.83	81.55	81.59	81.55	83.87	81.88
c, Å	58.56	58.49	58.48	58.57	58.26	58.84	58.72	58.40	58.59
α, °	90.00	90.00	90.00	90.00	90.00	90.00	90.00	90.00	90.00
β, °	110.28	110.02	109.78	110.70	110.06	110.78	110.32	109.01	110.54
γ, °	90.00	90.00	90.00	90.00	90.00	90.00	90.00	90.00	90.00
Resolution range, Å*	46.36 – 2.05 (2.17 – 2.05)	32.34 – 2.10 (2.21 – 2.10)	43.45 – 2.15 (2.28 – 2.15)	47.05 – 1.85 (1.96 – 1.85)	31.99 – 2.00 (2.12 – 2.00)	46.68 – 1.80 (1.90 – 1.80)	45.64 – 1.85 (1.96 – 1.85)	46.12 – 2.15 (2.28 – 2.15)	46.53 – 1.90 (1.95 – 1.90)
R_{sym}, %*	6.5 (48.0)	5.6 (19.1)	9.6 (47.9)	5.1 (41.4)	5.5 (48.6)	4.7 (38.7)	8.7 (45.8)	7.2 (44.8)	8.5 (43.9)
$I/\sigma I$*	10.62 (2.48)	16.7 (6.2)	13.07 (3.34)	13.01 (2.53)	16.52 (3.65)	13.85 (3.03)	7.84 (2.22)	15.41 (3.66)	8.53 (2.34)
Completeness, %*	99.4 (99.7)	97.2 (84.0)	97.9 (94.4)	98.5 (98.0)	98.7 (98.2)	99.1 (99.0)	99.2 (98.2)	99.3 (98.9)	99.1 (99.4)
Redundancy*	3.43 (3.49)	3.6 (3.4)	3.83 (3.73)	3.40 (3.38)	3.78 (3.80)	3.40 (3.46)	3.42 (3.39)	3.8 (3.8)	3.41 (3.37)
**Refinement**									
Resolution range, Å*	46.46 – 2.05	32.34 – 2.10	43.45 – 2.15	47.05 – 1.85	31.99 – 2.00	46.68 – 1.80	45.64 – 1.85	46.12 – 2.15	46.52 – 1.90
No. of reflections	30,288	53,009	25,808	41,649	32,303	44,615	40,985	27,693	36,078
R/R_{free}	0.198 / 0.227	0.176 / 0.229	0.181 / 0.229	0.179 / 0.213	0.180 / 0.237	0.197 / 0.222	0.207 / 0.249	0.183 / 0.241	0.196 / 0.234
$B_{factors}$, Å^2									
All	43.7	27.4	25.0	33.8	37.1	35.8	40.7	30.9	38.3
Protein	43.3	26.9	24.8	33.5	36.7	35.6	40.2	30.7	38.1
Ligands	66.3	26.2	22.2	31.2	45.1	39.5	42.0	31.6	41.4
Water	44.7	33.8	29.1	39.8	41.7	39.9	44.7	34.7	41.8
Rmsd									
Bond lengths, Å	0.004	0.005	0.005	0.007	0.006	0.003	0.004	0.004	0.004
Bond angles, °	1.145	0.737	0.736	0.967	0.973	0.752	0.764	0.717	0.745

*Values in parentheses are for the highest resolution shell.
